# Steatosis is involved in the progression of kidney disease in a high-fat-diet-induced non-alcoholic steatohepatitis mouse model

**DOI:** 10.1371/journal.pone.0265461

**Published:** 2022-03-16

**Authors:** Shintaro Hamada, Tomoaki Takata, Kentaro Yamada, Marie Yamamoto, Yukari Mae, Takuji Iyama, Suguru Ikeda, Tsutomu Kanda, Takaaki Sugihara, Hajime Isomoto

**Affiliations:** Division of Gastroenterology and Nephrology, Tottori University Faculty of Medicine, Yonago, Tottori, Japan; UCL Institute of Child Health, UNITED KINGDOM

## Abstract

Chronic kidney disease (CKD) and non-alcoholic steatohepatitis (NASH) are major health issues associated with the metabolic syndrome. Although NASH is a known risk factor of CKD, the mechanisms linking these two diseases remain poorly understood. We aimed to investigate alterations in the kidney complicated with dyslipidemia in an established NASH mouse model. Male C57BL6/J mice were fed with control diet or high-fat diet (HFD), containing 40% fat, 22% fructose, and 2% cholesterol for 16 weeks. Metabolic characteristics, histological changes in the kidney, endoplasmic reticulum (ER) stress, apoptosis, and fibrosis were evaluated by histological analysis, immunoblotting, and quantitative reverse transcription-polymerase chain reaction. Levels of serum aspartate aminotransferase, alanine aminotransferase, alkali-phosphatase, total cholesterol, and urinary albumin were significantly higher in mice fed with HFD. Remarkable steatosis, glomerular hypertrophy, and interstitial fibrosis were also shown in in the kidney by leveraging HFD. Furthermore, HFD increased the mRNA expression levels of *Casp3*, *Tgfb1*, and *Nfe2l2* and the protein level of BiP. We observed the early changes of CKD and speculate that the underlying mechanisms that link CKD and NASH are the induction of ER stress and apoptosis. Further, we observed the activation of *Nfe2l2* in the steatosis-induced CKD mouse model. This NASH model holds implications in investigating the mechanisms linking dyslipidemia and CKD.

## Introduction

Chronic kidney disease (CKD) is a public health burden that can be a risk for end-stage renal and cardiovascular diseases [1]. Diabetes mellitus is an important etiology in the development and progression of CKD and a major cause of end-stage renal disease in Japan [2]. Several agents targeting peptidyl hormones, such as dipeptidyl peptidase IV and glucagon-like peptide-1, or tubular reabsorption of glucose have recently been introduced [3–5]. These therapeutic options have largely modified the therapeutic strategies for improving diabetic kidney disease [6]. Moreover, obesity and dyslipidemia are also associated with the development of CKD [7]. Dyslipidemia worsens kidney injury by itself or in combination with diabetes mellitus. Although there are advancements in the management of diabetic kidney disease, limited therapeutic approaches are currently available for dyslipidemia. The development of the therapeutic approach for dyslipidemia will contribute to prevent CKD progression.

Non-alcoholic fatty liver disease and non-alcoholic steatohepatitis (NASH) are the most common liver diseases strongly associated with obesity and dyslipidemia. Patients with NASH are at high risk for developing CKD [8, 9]. Moreover, the severity of NASH is associated with the stage of CKD [10]. These findings suggest the existence of a common pathogenic mechanism underlying both conditions and interactions between NASH and CKD. The pathological findings observed in NASH are characterized by hepatic steatosis with inflammation and ballooning degeneration of hepatocytes [11]. Ectopic lipid accumulation in the liver plays an important role in inducing steatohepatitis and the progression of liver fibrosis. Recent investigations have also demonstrated steatosis—ectopic lipid deposition—in the renal tubules that further induce endoplasmic reticulum (ER) stress and apoptosis of the tubular cells [12]. Therefore, dysregulated lipid metabolism involved in the progression of “steatonephropathy” can be a therapeutic target for CKD treatment. Therefore, it is necessary to elucidate the mechanisms linking NASH and CKD at the molecular, cellular, and tissue levels. Recently, an ideal, diet-induced murine model expressing obesity, insulin resistance, and histologically proven NASH has been established [13]. In this study, we aimed to investigate the renal alterations complicated with dyslipidemia in this NASH mouse model. We revealed these alterations and consider that our study findings are instrumental in investigating the association between dyslipidemia and CKD.

## Materials and methods

### Animals and experimental design

Thirteen 12-week-old male C57BL6/J mice were obtained for the experiment (CLEA Japan, Tokyo, Japan). The mice were housed in a stainless steel cage in a room maintained at a temperature of 24 ± 2°C and a humidity of 40–60% under a 12-h/12-h light/dark cycle with free access to water. Thirteen mice were randomly assigned to two groups: control diet (CD) group and a high-fat diet (HFD) group (n = 6 and 7, respectively). HFD containing 40% fat, 22% fructose, and 2% cholesterol (catalog number: D09100310N), and CD containing 10% fat without fructose and cholesterol (D09100304) were prepared (Research Diets, New Brunswick, NJ, USA). The mice were fed with each diet for 16 weeks, followed by individually housing them in metabolic cages to collect 24-h urine. After the metabolic studies, the mice were anesthetized with medetomidine (0.75 mg/kg), midazolam (4 mg/kg), and butorphanol (5 mg/kg) and were then sacrificed; plasma was obtained by centrifugating cardiac blood for 20 min at 2,000 × *g*. After the cervical dislocation, kidneys were harvested and stored at −80°C or fixed for histological analysis. Urine and plasma were stored at −80°C until usage.

This study was carried out in strict accordance with the recommendations in the Guide for the Care and Use of Laboratory Animals of the National Institutes of Health. The protocol was approved by the Committee on the Ethics of Animal Experiments of the Tottori university (Protocol Number: 19-Y-39). All surgery was performed under sodium pentobarbital anesthesia, and all efforts were made to minimize suffering.

### Histological analysis

Harvested kidneys were fixed in 4% paraformaldehyde (Wako Pure Chemical Industries, Osaka, Japan), and then embedded in paraffin. Paraffin-embedded kidney sections (4 μm thick; cut with a microtome; TU-213, Yamato, Saitama, Japan) were used for histological analysis. The sections were dewaxed in xylene and stained with periodic acid-Schiff, Masson trichrome, and Oil-Red. Glomerular size, evaluated by the Bowman’s capsule area, and lipid droplets in the renal tubules, calculated as the relative area of droplets to the tubular area, were quantified in the periodic acid-Schiff-stained sections. Interstitial fibrosis was evaluated in the Masson trichrome-stained sections. Accumulations of neutral lipids were observed in glomeruli and renal tubules in Oil-Red-stained sections. Olympus BX51N microscope and Olympus DP25 camera (Olympus, Tokyo, Japan) were used for acquiring images. ImageJ software 1.53c (National Institute of Health, Bethesda, MD, USA) was used for the quantification.

### Quantitative reverse transcription-polymerase chain reaction (qRT-PCR)

The levels of mRNA expressions were quantified as previously described [14]. Briefly, harvested whole kidneys were homogenized with a pestle, followed by total RNA extraction using an RNeasy Mini Kit (catalog number: 217004, Qiagen, Hilden, Germany) according to the manufacturer’s protocol. RNA concentrations were measured spectrophotometrically using NanoDrop 1000 (Thermo Fisher Scientific, Tokyo, Japan), and 2 μg of total RNA was used for reverse transcription with a High-Capacity cDNA Reverse Transcription Kit (4374966, Thermo Fisher Scientific Inc., Tokyo, Japan) and iCycler Thermal Cycler (Bio-Rad Laboratories, Hercules, CA, USA): 25°C for 10 min, 37°C for 120 min, 85°C for 5 min. The resultant cDNA was subjected to qPCR. Thermal cycle reactions were performed in a Light Cycler 1.5 (Roche, Basel, Switzerland): a pre-incubation stage of 95°C for 10 min; an amplification stage of 95°C for 10 sec, 60°C for 10 sec, and 72°C for 10 sec for 45 cycles; the final step comprised cooling to 40°C for 30 sec. Primers used for the qPCR analysis are summarized in Table 1. *ACTB* was used as the internal control.

**Table 1:** List of primers used for qRT-PCR.

Gene Product	Forward Primer (5′-3′)	Reverse Primer (5′-3′)
*Hspa5*	GAG GCG TAT TTG GGA AAG AAG G	GCT GCT GTA GGC TCA TTG ATG
*Casp3*	TGA CTG GAA AGC CCG AAA CTC	GCA AGC CAT CTC CTC ATC AG
*Colla1*	CAT AAA GGG TCA TCG TGG CT	TTG AGT CCG TCT TTG CCA G
*Nfkb1*	ATT CCG CTA TGT GTG TGA AGG	GTG ACC AAC TGA ACG ATA ACC
*Ccl2*	AGG TCC CTG TCA TGC TTC TG	TCT GGA CCC ATT CCT TCT TG
*Tgfb1*	TGG AGC AAC ATG TGG AAC TC	GTC AGC AGC CGG TTA CCA
*Nfe2l2*	CCT CAC CTC TGC TGC AAG TA	GCT CAT AGT CCT TCT GTC GCT
*Keap1*	ATG TTG ACA CGG AGG ATT GG	TCA TCC GCC ACT CAT TCC T

### Western blot analysis

Proteins were extracted from the homogenized kidney tissues in freshly prepared 200 μL RIPA buffer (FUJIFILM, Tokyo, Japan) containing 50 mM Tris-HCl (pH 8.0) and 150 mM sodium chloride with protease and phosphatase inhibitor, cOmplete^TM^ ULTRA Tablets, and PhosSTOP^TM^ (Roche Diagnostics, Tokyo, Japan). Concentrations of the extracted proteins were determined using Pierce 660-nm Protein Assay Reagent (Thermo Fisher Scientific) according to the manufacturer’s instructions. Samples of 30 μg worth of protein were mixed with 4 × Laemmli buffer and 2-mercaptoethanol followed by heating at 95°C for 5 min. Thirty micrograms of the denatured protein was subjected to 10% sodium dodecyl sulfate-polyacrylamide gel electrophoresis. The proteins were subsequently transferred to a nitrocellulose membrane (GVS North America, Sanford, USA). Further, the membranes were blocked with 5% skim milk in tris-buffered saline. The membrane was incubated with rabbit monoclonal GRP78 antibody (ab21685; 1:1000, Abcam, Cambridge, U.K.) or rabbit anti-β-actin (13E5; 1:1000; CST; Tokyo, Japan) overnight at 4°C. The membranes were then washed with tris-buffered saline followed by incubation with horseradish peroxidase-conjugated secondary antibodies (ab97051; 1:2000; Abcam, Cambridge, U.K.) for 1 h at 25°C. Signals were visualized by Clarity Western ECL Substrate (catalog number: 1705061, Bio-Rad Laboratories, Tokyo, Japan) and an image analyzer (LAS-3000 mini; Fujifilm, Tokyo, Japan). Rabbit β-actin was used to normalize the signals.

### Statistical analysis

Comparison between the groups was assessed using unpaired *t*-test. P-values less than 0.05 were considered as statistically significant. GraphPad Prism 7.0. for Windows (GraphPad Software, San Diego, CA, USA) was used for the analysis.

## Results

### Biochemical analysis

We found significant difference in the body weight after 16 weeks of diet between the two groups. Serum transaminases, including aspartate aminotransferase, alanine aminotransferase, and alkaline-phosphatase, total cholesterol, and urinary albumin adjusted by urinary creatinine were significantly higher in the HFD-fed mice than in the CD-fed mice. Surprisingly, we observed significant decreases in serum triglyceride levels and urinary sodium excretion in the HFD-fed mice (Table 2).

**Table 2:** Metabolic parameters of the two groups.

Parameter	Control Diet	High-Fat Diet	P-Value
Body weight (g)	31.7 ± 1.4	47.9 ± 2.0	<0.001
Urine volume (mL/d)	1.4 ± 0.8	1.2 ± 0.5	0.683
Cr clearance (mL / min)	0.32 ± 0.15	0.27 ± 0.08	0.484
Blood glucose (mg/dL)	389.8 ± 112.5	509.6 ± 103.6	0.074
AST (IU/L)	91.0 ± 50.9	264.4 ± 69.0	<0.001
ALT (IU/L)	42.7 ± 39.5	325.9 ± 87.7	<0.001
ALP (IU/L)	238.8 ± 24.9	624.4 ± 91.3	<0.001
Total cholesterol (mg/dL)	72.3 ± 7.0	309.7 ± 30.0	<0.001
Triglyceride (mg/dL)	76.5 ± 17.4	17.1 ± 6.5	<0.001
Urinary sodium (mmol/day)	0.41 ± 0.15	0.16 ± 0.06	0.006
Urinary Alb/Cr (μg/g/Cr)	0.23 ± 0.09	0.36 ± 0.08	0.024

AST, aspartate aminotransferase; ALT, alanine aminotransferase; ALP, alkali-phosphatase; Alb, albumin; Cr, creatinine. Data are shown as mean ± SD.

### Histological analysis of renal alterations

**Fig 1:**
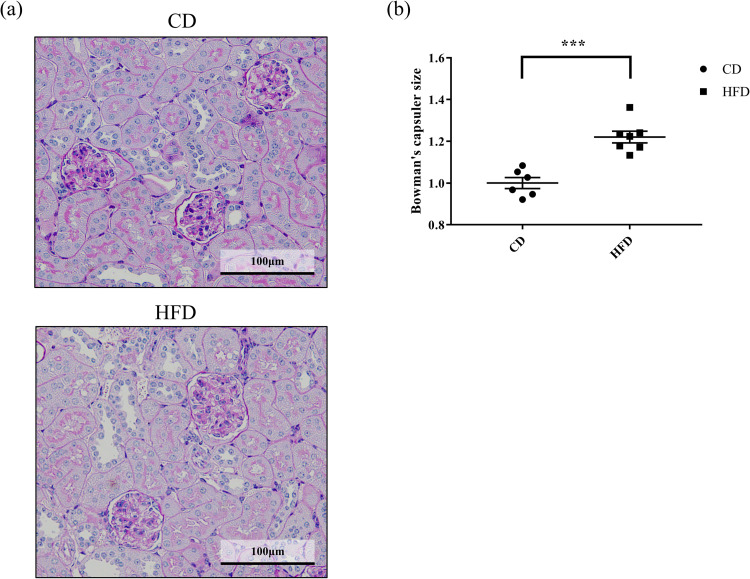
Effect of HFD on glomerular size. (a) Representative images of periodic acid–Schiff-stained C57BL6/J mouse renal tissue sections. (b) Quantification of the size of glomeruli. Results are expressed relative to Bowman’s capsule area. At least 20 glomeruli from the all mice are quantified. Bars indicate mean ± SEM. *** p < 0.001 (unpaired t-test). n = 6 in CD and 7 in HFD, respectively. CD, control diet group; HFD, high-fat diet group.

**Fig 2:**
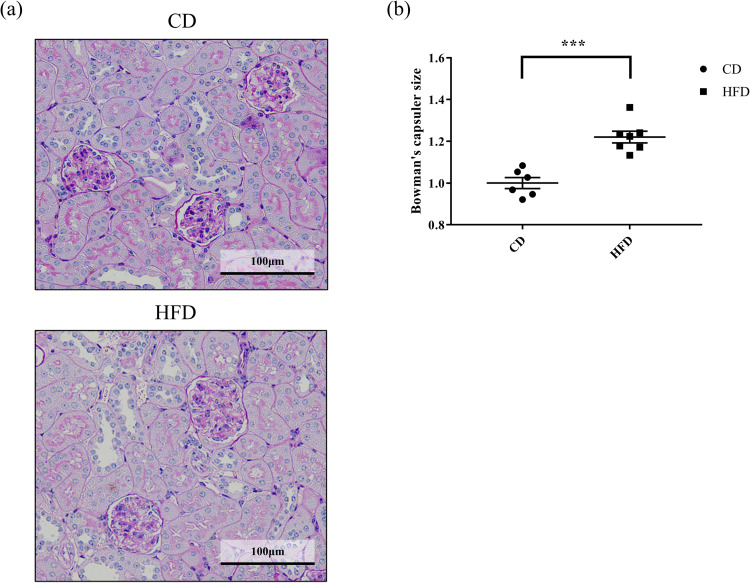
Effect of HFD on lipid deposition in renal tubules. Representative images of periodic acid–Schiff-stained C57BL6/J mouse renal tissue sections. Mice are fed with (a) CD or (b) HFD for 16 weeks. Lipid droplets are observed in the tubular epithelial cells (arrowheads) in renal tissues of HFD-fed mice. The droplets are devoid in the CD-fed mice. (c) Quantification of the fractional area of lipid droplets. Ratio of the total area of lipid droplets to the tubular area is quantified at randomly captured images from the all mice. Bars indicate mean ± SEM. *** p < 0.001 (unpaired t-test). n = 6 in CD and 7 in HFD, respectively. CD, control diet group; HFD, high-fat diet group.

**Fig 3:**
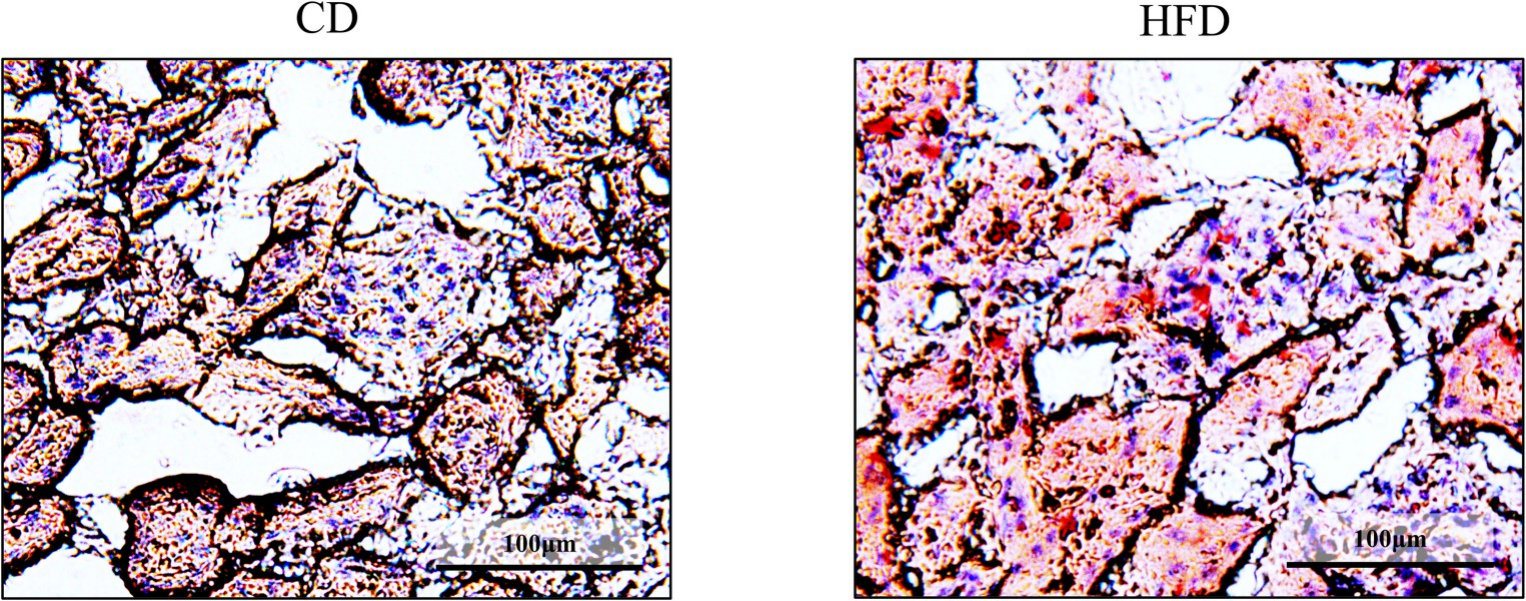
Oil-Red staining of the kidney. Representative images of Oil-Red-stained C57BL6/J mouse renal tissue sections. Lipid depositions are stained in red. Accumulations of neutral lipids are observed in glomeruli and renal tubules in HFD-fed mice. CD, control diet group; HFD, high-fat diet group.

**Fig 4:**
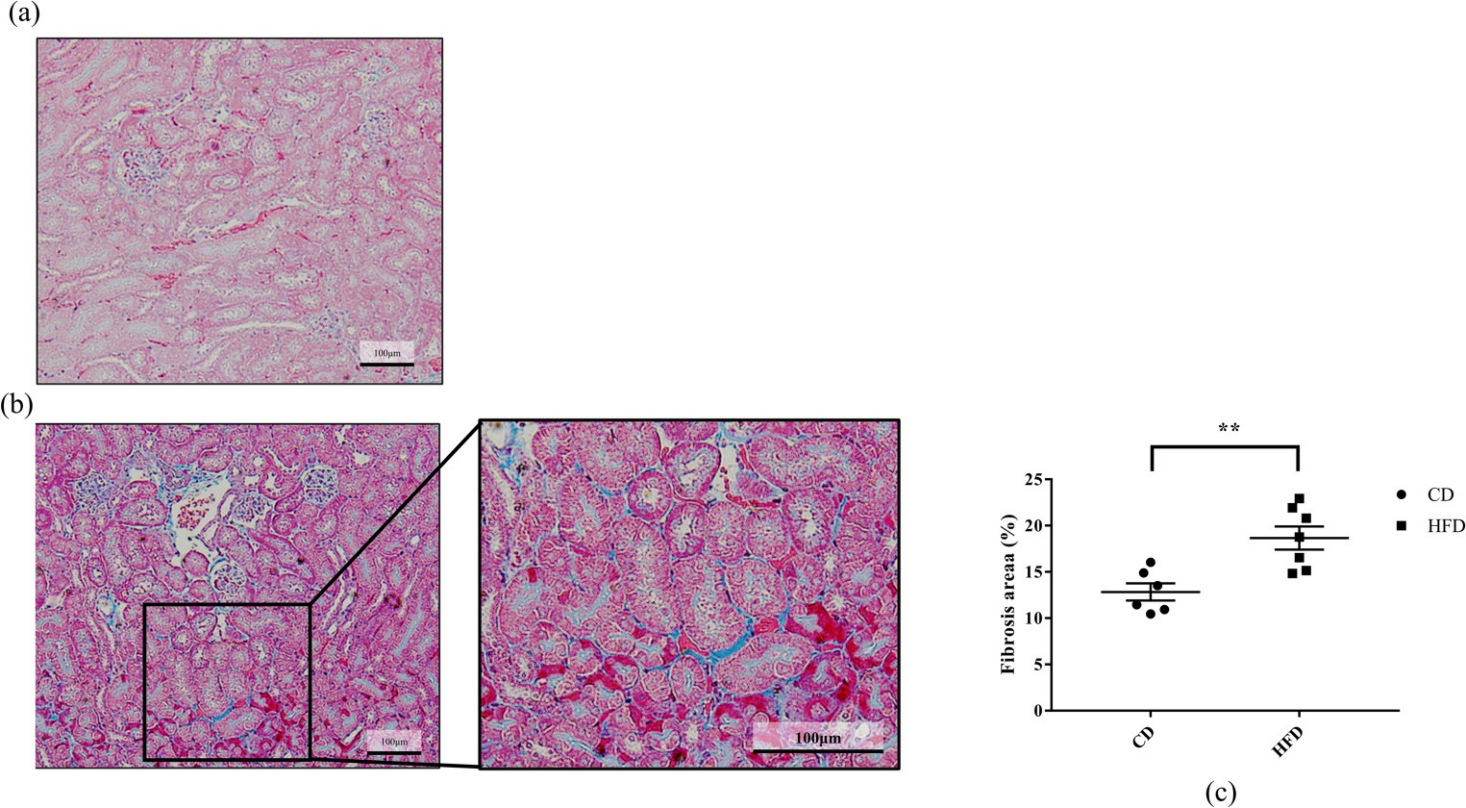
Effect of HFD on interstitial fibrosis. (a) Representative images of Masson trichrome-stained C57BL6/J mouse renal tissue sections. Mice are fed with (a) CD or (b) HFD for 16 weeks. (c) Quantification of the area of fibrosis. Results are expressed as the percentage of fibrotic area to the whole tissue area. The quantification is based on randomly captured five fields from the all mice. Bars indicate mean ± SEM. ** p < 0.01 (unpaired t-test). n = 6 in CD and 7 in HFD, respectively. CD, control diet group; HFD, high-fat diet group.

Effect of HFD on histological changes, including glomerular hypertrophy, ectopic lipid deposition in the renal tubules, and interstitial fibrosis, were evaluated. Consequently, we observed that the Bowman’s capsule was significantly larger in the HFD group, indicating that HFD induced glomerular hyperfiltration (Fig 1). Further, HFD-fed mice had remarkable lipid droplet formation in their renal tubules, which were scarce in the CD-fed mice, as observed by periodic acid-Schiff staining (Fig 2). This observation was confirmed in the Oil-Red-stained tissue sections, wherein significant accumulation of neutral lipids in the glomerular and tubulointerstitial cells were observed in HFD-fed mice, as compared to that in CD-fed mice (Fig 3). These changes were accompanied by a significantly high rate of fibrosis in the HFD-fed mice, as demonstrated by Masson trichrome staining (Fig 4).

### Gene expression analysis

**Fig 5:**
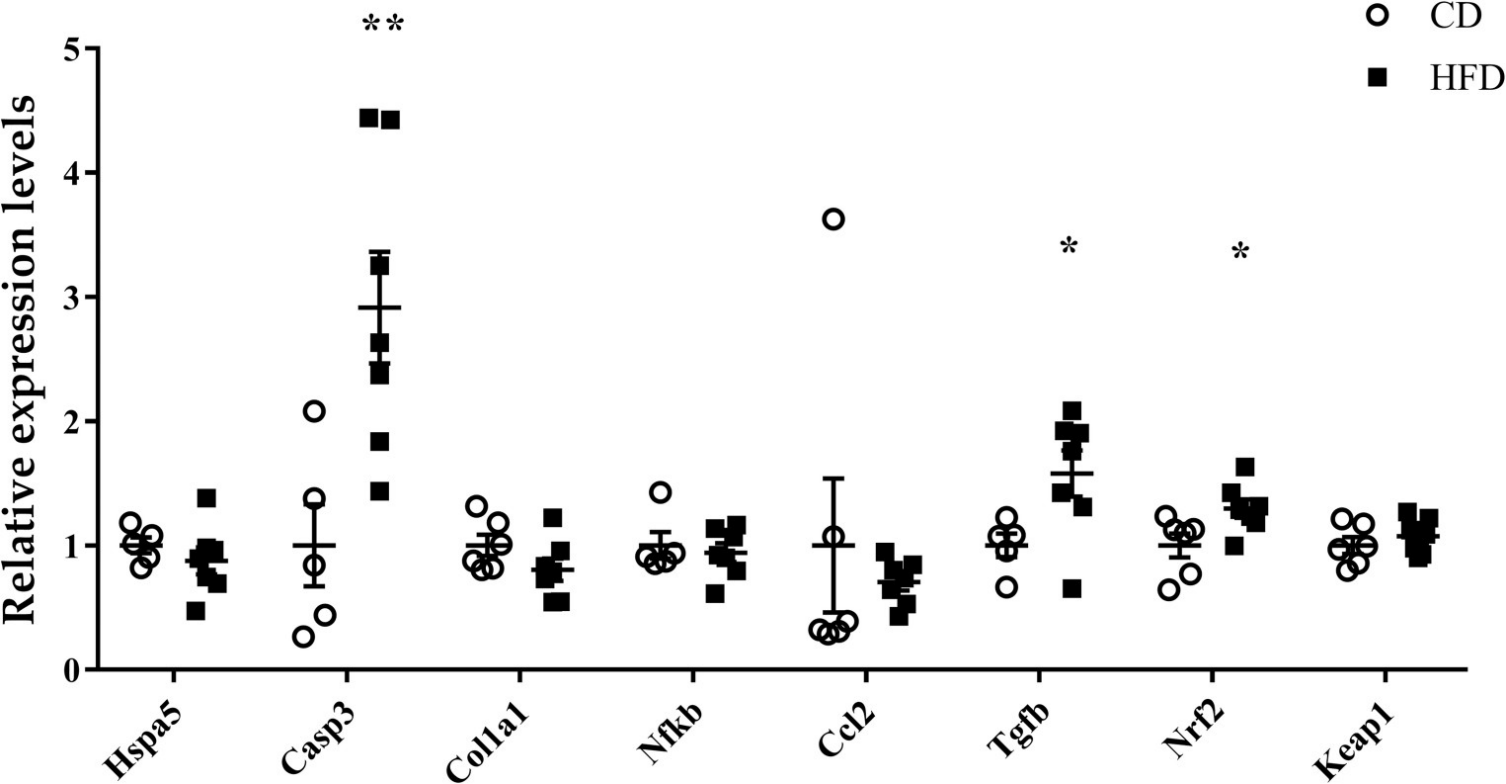
Gene expression analyses. Relative gene expression levels of *Hspa5*, *Casp3*, *Col1a1*, *Nfkb1*, *Ccl2*, *Tgfb1*, *Nfe2l2*, and *Keap1* analyzed by quantitative reverse transcription-polymerase chain reaction (qRT-PCR) in C57BL6/J mouse renal tissue homogenates. *ACTB* is the internal control. The expression levels are presented relative to the control group. Bars indicate mean ± SEM. * p < 0.05; ** p < 0.01 (unpaired t-test). n = 5 or 6 in CD and 7 in HFD, respectively. CD, control diet group; HFD, high-fat diet group.

To determine if the increase in lipid droplets affected inflammation, ER stress, apoptosis, and fibrosis in the kidney, we performed qRT-PCR. We observed that the expression levels of caspase 3 (*Casp3*), transforming growth factor, beta 1 (*Tgfb1*), and nuclear factor, and erythroid derived 2, like 2 (*Nfe2l2*) significantly increased in the HFD group. However, those of heat shock protein 5 (*Hspa5*), collagen, type I, alpha 1 (*Col1a1*), nuclear factor of kappa light polypeptide gene enhancer in B cells 1, p105 (*Nfkb1*), chemokine (C-C motif) ligand 2 (*Ccl2*), and kelch-like ECH-associated protein 1 (*Keap1*) were not significantly different between the two groups (Fig 5).

### Western blot analysis

**Fig 6:**
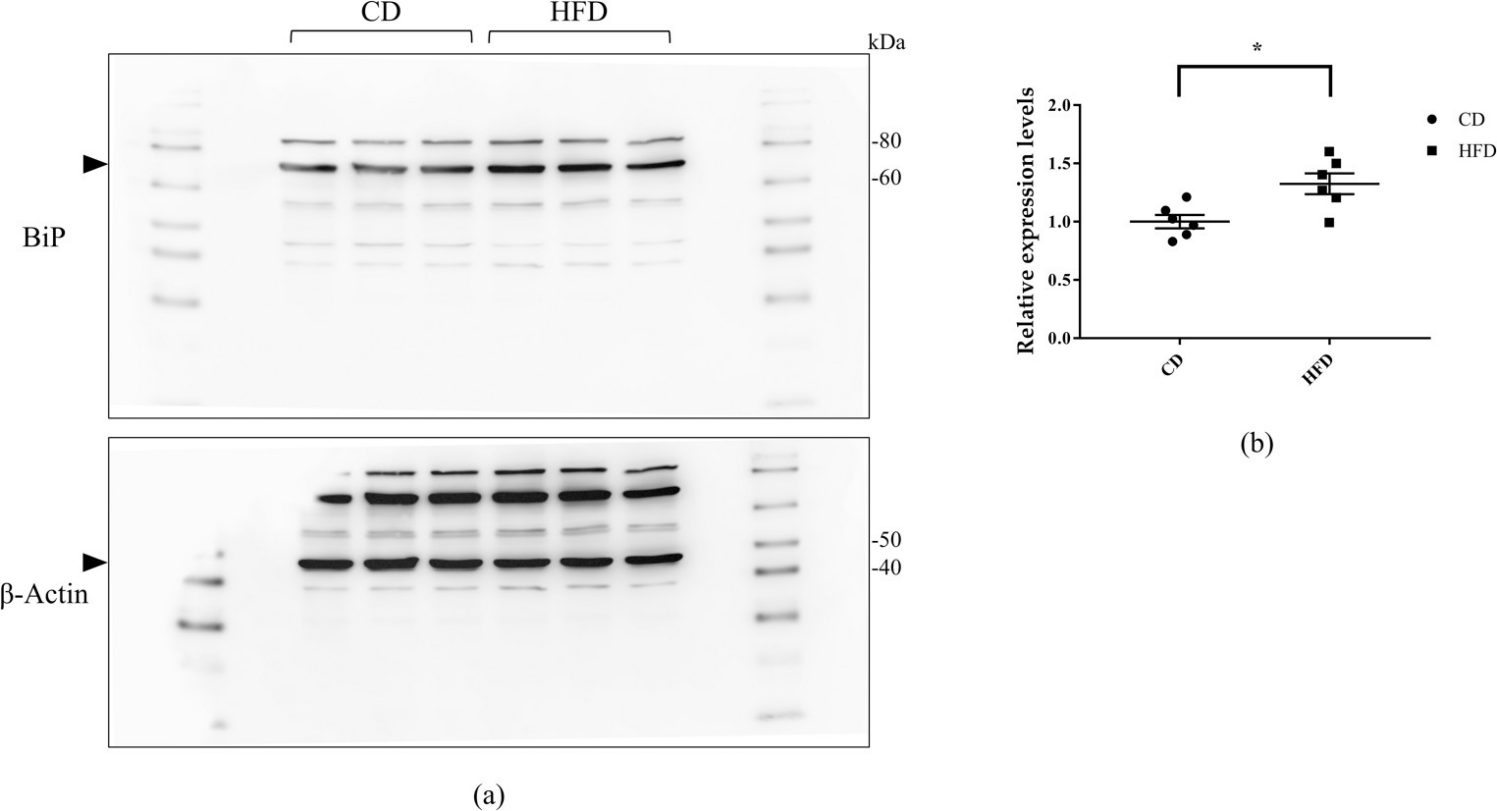
Western blot analysis. (a) Representative images of immunoblots for BiP in C57BL6/J mouse renal tissue homogenates. (b) Quantification of the signal intensities, expressed relative to the control group. β-actin is the loading control. Bars indicate mean ± SEM. * p < 0.05 (unpaired t-test). n = 6 each group. CD, control diet group; HFD, high-fat diet group.

Since ER stress is involved in the mechanism of steatonephropathy, we further analyzed the protein expression of BiP, a master regulator of ER stress. Although its gene expression levels did not differ between the groups, protein expression of BiP was significantly high in the HFD group (Fig 6).

## Discussion

In the present study, we investigated alterations in kidneys complicated with dyslipidemia in an established NASH mouse model. Remarkable steatosis, glomerular hypertrophy, and interstitial fibrosis together with increased expression of genes associated with the development of apoptosis and fibrosis were observed.

A number of NASH and CKD animal models that represent these metabolic syndromes individually have been established and described previously. Reports on the mechanisms linking dyslipidemia and CKD are scarce. In addition, comprehensive structural and histopathological renal alterations in NASH model mice have not been previously investigated. Therefore, an in vivo model can help to better understanding the mechanisms linking CKD and NASH and thereby, to elucidate NASH-induced alterations in the kidney. Traditional murine models used to study hepatic fibrosis often include choline- and methionine-deficient diets or carbon tetrachloride-induced models [15–17]. Not just induced ones but genetically modified models have also been widely investigated to study hepatic fibrosis [18, 19]. However, these models do not represent the entire spectrum of the pathophysiologies seen in human NASH. The same applies to murine models for CKD. Further, quite a few genetically modified murine models, such as *ob/ob*, Akita, and MKR mouse, that mimic metabolic disorders have been established [20–22]; however, these are not ideal to reflect renal alterations caused by metabolic syndromes. Therefore, we considered that an ideal model to recapitulate human NASH and steatosis-induced CKD would be achieved by leveraging a high-fat containing diet. Although HFD-fed mouse showed obesity, renal lipid deposition, and glomerular structural changes [23, 24], involvement of liver was not confirmed in this model. Because there are two clusters of phenotypes with a simple steatosis in the liver and with a progression to steatohepatitis, it is worth investigating renal involvement in steatohepatitis. Fructose accelerates lipid synthesis at a much higher rate than other carbohydrates [25], and was used to induce NASH [26]. It has been revealed that the diet containing high fat, high fructose and high cholesterol was suitable to induce NASH compared to high-fat alone, based on the findings that diet containing high fat, fructose and cholesterol showed increased macrophage infiltration and fibrosis in the liver [27, 28]. Excessive fructose affected the circulating lipid pool and the combination of high-fat and high-fructose accelerated hepatic injury than high-fat alone [28]. In addition, excessive consumption of fructose induced inflammation and fibrosis in the kidney as well [29]. Therefore, fructose contained in the diet potentially accelerate the kidney injury. We utilized a diet containing high-fat and fructose, an established diet to induce NASH in the present study.

We observed a significant decrease in serum triglyceride levels in mice fed with HFD. This unexpected finding is concurrent with a previous report that rendered impairment in secretion of very low-density lipoprotein from the liver as the underlying reason [26]. The phenotype of hepatic lipid accumulation is related to the type of the contained fat because trans fats causes the suppressed triglyceride secretion from the liver [30]. Since NASH and dyslipidemia is strongly associated with each, it has been difficult to clearly distinguish the direct association between NASH and CKD. Our diet-induced mouse model has strong phenotype in the liver, supporting in part the direct link between NASH and CKD. Further, we observed that the mice fed with HFD showed remarkable histological alterations in the glomeruli and renal tubules. These changes were accompanied with increased ER stress, apoptotic signals, and fibrosis. Reportedly, lipid deposition in renal tubules is associated with increased ER stress in genetically modified obese mice [12]. We observed the diet-induced steatosis and ER stress in the kidney by leveraging HFD. ER stress directly activates caspase cascade pathway leading to increased apoptotic signals [31–33]. We speculate that lipid deposition in the renal tubules induced ER stress resulting in apoptosis by the activation of the caspase pathway. We know that TGF-β is a multifunctional cytokine that induces tissue fibrosis and epithelial-mesenchymal transition [34]. Recent *in vitro* studies using lung fibroblasts and mesangial cells have revealed that ER stress caused the activation of the TGF-β pathway followed by fibrosis [35–37]. Our observation is in line with these findings; elevated ER stress led to an increased expression of TGF-β and thereby renal fibrosis.

Previous studies have reported the mechanisms of inflammation in hepatocyte and liver fibrosis. Inflammatory cytokines, such as TNF-α, IL-6, or MCP-1, led to the induction of NFkB that accelerates the progression of liver fibrosis [38, 39]. However, we did not see any significant difference in the mRNA expression levels of NFkB in the present study. This suggests a mechanism other than inflammatory response underlying the progression of renal fibrosis. Further, we observed higher levels of *Nfe2l2* expression in mice fed with HFD than in those fed with CD. Nfe2l2 is an antioxidant transcription factor that activates the transcription of antioxidant enzymes [40–42]. Reportedly, Nfe2l2 significantly increases in the kidney after three months of diabetes, followed by a significant reduction at six months, indicating initial upregulation [43]. Similar observations have been reported in other organisms and these findings suggest that Nfe2l2 increases as the initial response to oxidative stress in diabetes to overcome injury [44]. We speculate that four months of HFD feeding caused the early upregulation of Nfe2l2 response.

In the present study, the NASH mouse model exhibited steatosis in the kidney along with interstitial fibrosis. Although we could observe a possible association of ER stress and fibrosis underlying steatosis-induced CKD, further investigations are required to uncover better and in depth understanding of the underlying mechanisms.

There are some limitations to this study. We observed the initial upregulation of Nfe2l2 in mice fed with HFD for 16 weeks, but we could not confirm the later reduction because that needed one more mouse group fed with HFD for longer than them. Second, the differences in kidney injury in mice fed with high-fat alone and those fed with high-fat / high-fructose remain to be clarified. However, surplus amount of fructose is evidently associated with kidney injury and the combination of high-fat and high-fructose intensified the liver inflammation and fibrosis. Therefore, we speculate that HFD used in our study potentially exacerbated the kidney injury in our study. We validated the renal alterations in a prominent diet-induced NASH model.

We consider that the NASH mouse model offers a better understanding of the mechanisms underlying steatosis-induced kidney injury, and therefore, holds implications for developing novel and effective therapeutics.

## Conclusions

In conclusion, we observed remarkable steatosis, glomerular hypertrophy, and interstitial fibrosis in mice fed with high fat and fructose. We consider that the underlying mechanisms are induction of ER stress and apoptotic signals. Furthermore, we observed that the steatosis-induced CKD mouse model showed the activation of Nfe2l2, an antioxidant transcription factor. Our study indicates that the diet-induced NASH mouse model has advantages over conventional models for investigating the mechanisms linking NASH and CKD. This NASH mouse model can be useful for the development of therapeutics for major renal and hepatic metabolic disorders.

## Supporting information

S1 Fig (TIF)
