# Correction: Steatosis is involved in the progression of kidney disease in a high-fat-diet-induced non-alcoholic steatohepatitis mouse model

**DOI:** 10.1371/journal.pone.0329654

**Published:** 2025-08-04

**Authors:** The PLOS One Staff

The images for Fig 2 is the same as Fig 1. The Fig 2 caption is correct. The authors have provided a corrected version of Fig 2 here.

**Fig 2:**
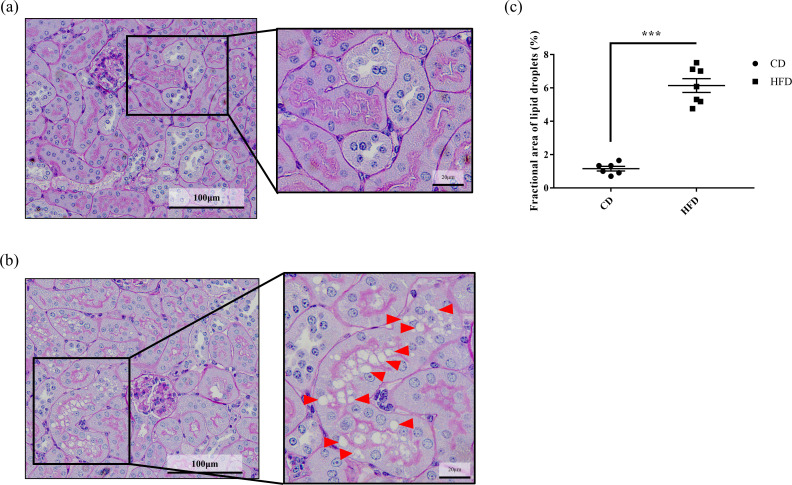
Effect of HFD on lipid deposition in renal tubules. Representative images of periodic acid–Schiff-stained C57BL6/J mouse renal tissue sections. Mice are fed with **(a)** CD or **(b)** HFD for 16 weeks. Lipid droplets are observed in the tubular epithelial cells (arrowheads) in renal tissues of HFD-fed mice. The droplets are devoid in the CD-fed mice. **(c)** Quantification of the fractional area of lipid droplets. Ratio of the total area of lipid droplets to the tubular area is quantified at randomly captured images from the all mice. Bars indicate mean ± SEM. *** p < 0.001 (unpaired t-test). n = 6 in CD and 7 in HFD, respectively. CD, control diet group; HFD, high-fat diet group.

The publisher apologizes for the error.
