# Supplementary figures and images for: Steatosis is involved in the progression of kidney disease in a high-fat-diet-induced non-alcoholic steatohepatitis mouse model
Source: PLoS One. 2022 Mar 16;17(3):e0265461. doi: 10.1371/journal.pone.0265461 (PMC8926260; doi:10.1371/journal.pone.0265461)

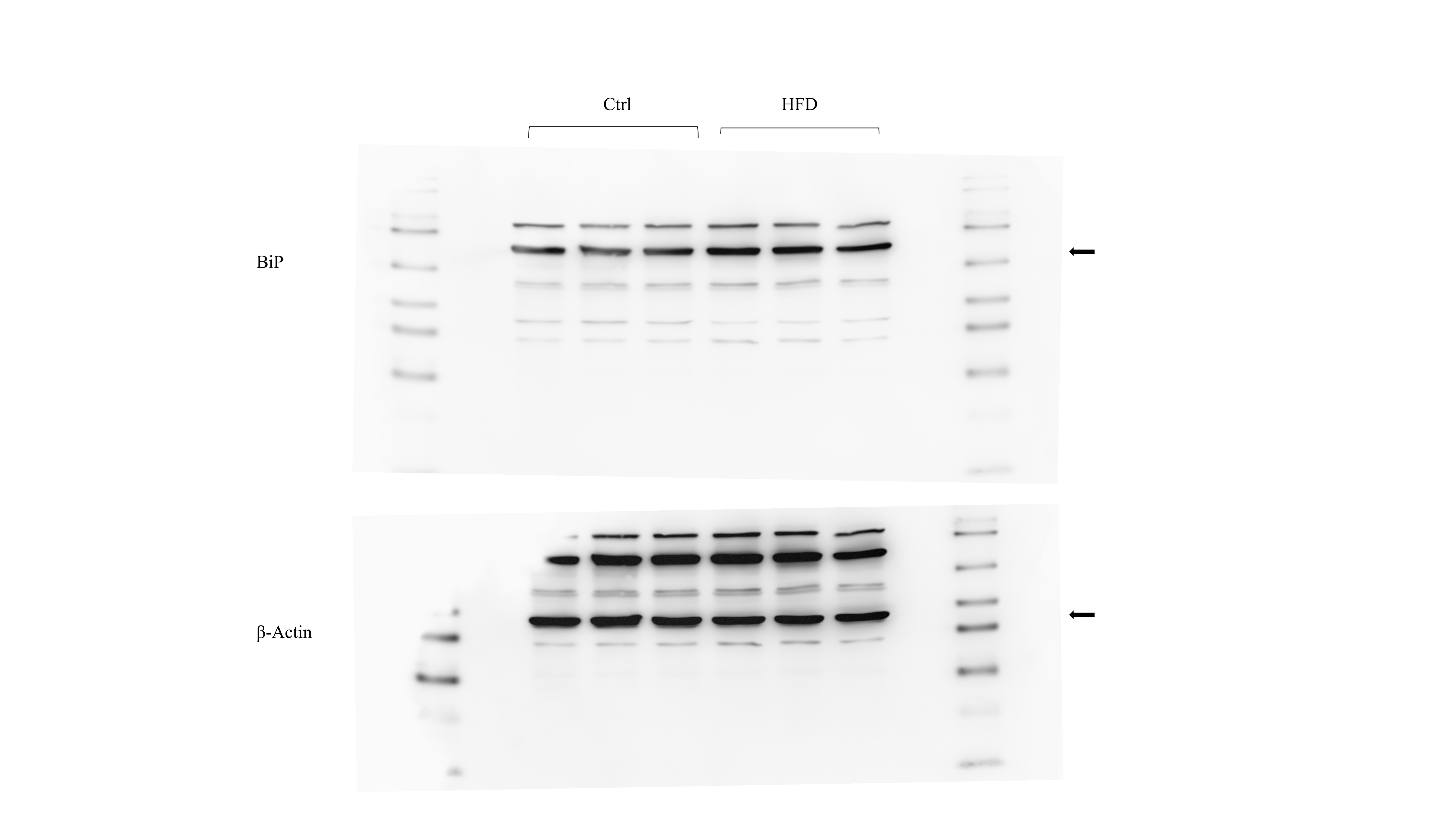

Supplement: S1 Fig — (TIF) [file pone.0265461.s001.tif]
